# Thermosensitive Hydrogels and Advances in Their Application in Disease Therapy

**DOI:** 10.3390/polym14122379

**Published:** 2022-06-12

**Authors:** Ranran Fan, Yi Cheng, Rongrong Wang, Ting Zhang, Hui Zhang, Jianchun Li, Shenghan Song, Aiping Zheng

**Affiliations:** 1 School of Pharmacy, Bengbu Medical College, Anhui 233030, China; frr0118@163.com; 2 College of Pharmacy, Yanbian University, Jilin 133002, China; cy17804330272@163.com; 3 School of Pharmacy, North China University of Science and Technology, Hebei 063210, China; wrr199608@163.com; 4 School of Basic Medical Sciences, Zhengzhou University, Zhengzhou 450001, China; ztandwyk@163.com; 5 Institute of Pharmacology and Toxicology, Academy of Military Medical Sciences, Academy of Military Sciences, Beijing 100850, China; apzheng@163.com; 6 Department of Vascular Surgery, Beijing Chaoyang Hospital, Capital Medical University, Beijing 100020, China

**Keywords:** thermosensitive hydrogel, local administration, drug release mechanism, drug delivery, disease treatment

## Abstract

Thermosensitive hydrogels, having unique sol–gel transition properties, have recently received special research attention. These hydrogels exhibit a phase transition near body temperature. This feature is the key to their applications in human medicine. In addition, hydrogels can quickly gel at the application site with simple temperature stimulation and without additional organic solvents, cross-linking agents, or external equipment, and the loaded drugs can be retained locally to improve the local drug concentration and avoid unexpected toxicity or side effects caused by systemic administration. All of these features have led to thermosensitive hydrogels being some of the most promising and practical drug delivery systems. In this paper, we review thermosensitive hydrogel materials with biomedical application potential, including natural and synthetic materials. We describe their structural characteristics and gelation mechanism and briefly summarize the mechanism of drug release from thermosensitive hydrogels. Our focus in this review was to summarize the application of thermosensitive hydrogels in disease treatment, including the postoperative recurrence of tumors, the delivery of vaccines, the prevention of postoperative adhesions, the treatment of nervous system diseases via nasal brain targeting, wound healing, and osteoarthritis treatment.

## 1. Introduction

How a drug enters the body affects its absorption, distribution, metabolism, and excretion. However, the therapeutic effect is directly related to its transmission mode [1]. Oral and vascular administrations of drugs are the most common drug therapy routes for diseases because of their advantages, such as convenience, effectiveness, and patient friendliness [2]. However, these two methods of administration are distributed through the whole-body blood through the circulation system to individual organs and parts. Only a small part of the drug reaches the diseased or targeted part, and most drugs spread to the parts where they are not needed. For normal tissues or organs, especially the treatment of tumor-related disease, this may result in unexpected damage [3,4,5]. Therefore, local treatment has attracted extensive attention: obvious advantages include avoiding liver metabolism, the highest concentrations directly entering the lesions, reducing the side effects of drugs, easy application, reducing the number of drugs, and improving patient compliance [6,7,8].

Local release allows the maximum concentration of the drug molecules to be transmitted nearby the target, thereby reducing the dosage and drug toxicity caused by the use of a large number of drugs. Therefore, local administration is an attractive choice over system administration. In addition, with the gradual development and application of local treatment methods, researchers focused on the ability to use chemicals and carrier materials to deliver drugs. A current hotspot of local treatment application involves designing an injection or material that can be directly applied to and sprayed on the lesion to form a controllable local drug molecular library [9,10,11]. These materials can generally be divided into solid implants and injection materials. Specifically, solid implants refer to sponges, films, fibers, sticks, and brackets [12]. These need to be implanted at the target site through surgery. If the treatment is terminated, they need to be removed by secondary surgery. Injectable materials include nanoparticles or particles, in situ hydrogels, and their combinations. Drugs can be introduced to the target location through local injection. In most cases, no surgical removal is required, as the materials degrade in the body [13].

Hydrogels are aqueous, viscous, semisolid preparations consisting of a gel matrix and a drug. Hydrogels are popular for local administration because they can continuously release a drug at a given site. In addition, hydrogels are widely used in drug delivery, tissue engineering, and biosensors, among other applications [14]. In water media, the hydrogel is prepared by physical or chemical crosslinking [14,15,16]. Chemical crosslinked hydrogel can be formed by the Michael addition reaction, photopolymerization, enzymatic reaction, or the formation of disulfide bonds [15,16]. The physical linkage of hydrogels involves the formation by hydrophobic interactions under the environmental stimulation of the water-soluble polymer chain [17,18]. However, physical crosslinked hydrogels provide more advantages because they do not require a crosslinking agent [19]. The most common hydrogel fabrication strategy is physical crosslinking between chain polymers, such as hydrogen bonds, π–π stacking, dipole–dipole interactions, and hydrophobic interactions, which are usually responsible for molecular self-assembly in hydrogels [20,21]. These stimulated physical crosslinked hydrogels are considered a smart drug transmission system that can control the timing and location of drug release, effectively ensuring drug stability without degradation [22].

**Figure 1:**
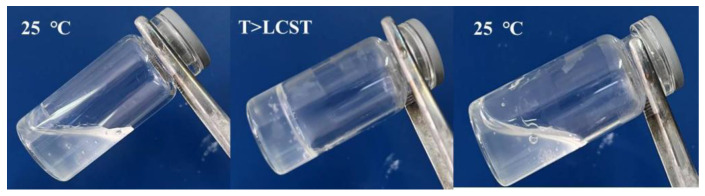
Digital photos of samples at room temperature (25 °C), at or above gelling temperature (T > LCST), and at room temperature (25 °C) (In the example, the thermosensitive gel was composed of poloxamer 407, poloxamer 188, and carbomer).

In general, stimulation causes changes in the local properties of a polymer. The most commonly used stimuli are pH, temperature, oxidation potential, light excitation, and enzyme changes. Polymer hydrogels are usually low-viscosity liquids. After the external stimulation of a polymer, the drug site changes and the polymer becomes a semisolid gel, which can be restored to the initial state after stimulation [23] (Figure 1). Among the many stimulating reactions, polymers that rapidly react to local temperature changes are called temperature-responsive polymers and have attracted considerable interest in the biomedical field. A temperature-responsive hydrogel system allows the in situ formation of gel, which can be used to simply transport drugs to the target site and can be liquid at environmental temperature and can solidify at the elevated physiological temperature in the body. In certain systems, once the required temperature is reached, the hydrogel immediately forms, which is called the sol–gel transition [24]. However, temperature-sensitive systems where the temperature is the only stimulus are some of the mildest methods and do not require other chemical initiators or enzymatic reactions [25], which leads to an easier and more economical process [26,27]. In recent years, thermosensitive hydrogels have become promising drug carriers due to their biological degradation, low toxicity, high load, site specificity, and sustained drug release [28].

Thermosensitive hydrogels are a hotspot research object in the field of biomedical science and engineering, especially in the field of drug delivery and tissue engineering [29]. As such, in this paper, we briefly review the characteristics, gel-forming mechanism, common materials, drug release methods, and local applications of thermosensitive hydrogels, including postoperative recurrence of tumors, delivery of vaccines, prevention of postoperative adhesions, treatment of nervous system diseases via nasal brain targeting, wound healing, and treatment of osteoarthritis.

## 2. Thermosensitive Hydrogel: A Brief Overview

**Figure 2:**
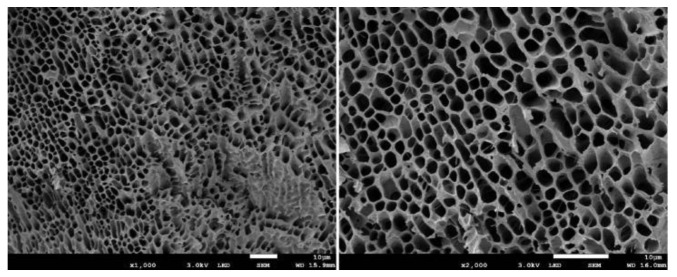
Three-dimensional network structure formed by hydrogel.

**Figure 3:**
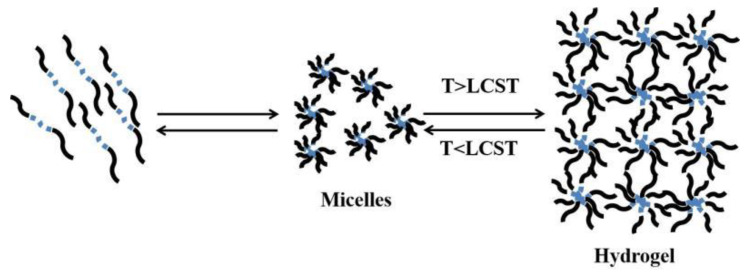
Schematic representation of the micellization and gel formation of aqueous solution. (LCST was defined as the lower critical solution temperature, UCST was defined as the upper critical solution temperature.)

Thermosensitive hydrogel is a kind of temperature-sensitive material, and the change of ambient temperature changes its physical state, resulting in the change of the sol–gel state. When stimulated by temperature, high-molecular polymers with temperature sensitivity change from a dispersed micelle state to dense a three-dimensional network structure (Figure 2). Phase separation occurs when the polymer solution is higher than or below a particular temperature (critical dissolution temperature, CST). This phase separation is due to the hydrophobic effect between polymer chains, so the polymer self-assembly and aggregation in aqueous solution to form a hydrogel [30] (Figure 3).

**Figure 4:**
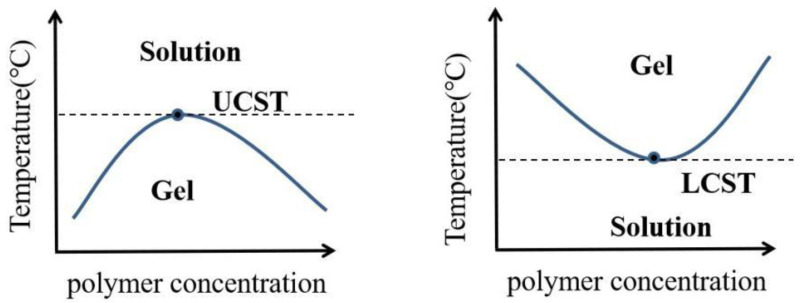
LCST-type formulation undergoes sol–gel transition with increase in temperature while UCST-type formulation undergoes sol–gel transition as the temperature decreases.

The low and high critical dissolution temperatures during phase separation are represented by LCST and UCST, respectively (Figure 4). LCST was defined as the lower critical solution temperature (LCST), that is, the phase transition of thermally responsive polymers will occur in the environment above this temperature, and the material can be dissolved at a low temperature; when heated above the LCST, the molecules precipitate from the solution and undergo a sol–gel phase transition. UCST was defined as the upper critical solution temperature (UCST), that is, the phase transition of thermally responsive polymers will occur in the environment below this temperature, and the material cannot be dissolved at low temperature; when heated above UCST, the material will dissolve. Heat-response polymers with an LCST have good biomedical application prospects because these polymers can form hydrogels only above the LCST, which is closer to the temperature of the human body. Polymers with heat-response polysaccharides, including Kara gum, starch, agarose, and cold glue, have a UCST and cannot respond at physiological temperature [31]. The polymer can be soluble at the LCST, which occurs in liquid form. Shrinkage starts at the LCST, becoming hydrophobic and insoluble, forming a hydrogel. More specifically, polymers close to the critical temperature exhibit a phase change from soluble (random curl) to insoluble (crash or micelle form).

At the molecular level, the hydrophobic molecules of heat-responsive polymers interact at high temperatures and are exposed to the water molecules that collapse in the polymer chain, swelling to form a hydrogel [18]. Thermodynamically, phase separation transition in the sol–gel state is relocated due to freedom [32]. The association free-energy varies with enthalpy, entropy, and temperature (∆G = ∆H − T∆S) because the positive enthalpy term ∆H is smaller than the entropy term ∆S. An increase in temperature results in an increase in T∆S; a negative ∆G value favors polymer chain association [33,34]. Some molecular interactions, such as hydrogen bonds and hydrophobic effects, help with phase separation.

### 2.1. Common Temperature Sensitive Materials and Gelation Mechanism

Temperature-sensitive biomaterials are a new intelligent material for preparing thermosensitive hydrogels. Organic polymer materials are generally formed by hydrogen bonds and π–π, van der Waals, and hydrophobic interactions. The materials commonly used in thermosensitive hydrogels are generally divided into two categories: synthetic and natural. Synthetic materials include poloxamers [35,36,37,38], poly *N*-isopropylacrylamides [39,40,41,42], and polyethylene glycol [43,44,45]; naturally formed materials include chitosan [46,47,48,49] and cellulose [50,51,52].

**Figure 5:**
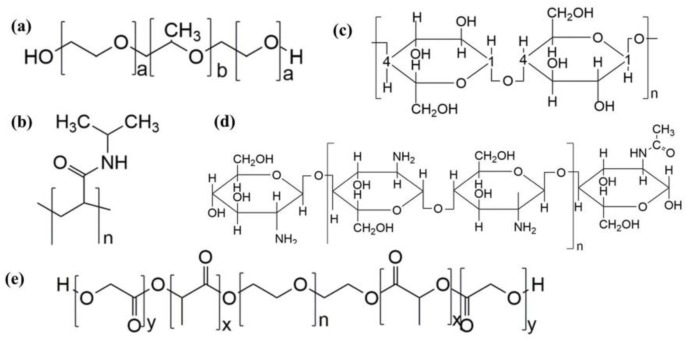
The structural formula of (**a**) poloxamer, (**b**) poly(*N*-isopropylacrylamide), (**c**) cellulose, (**d**) chitosan, and (**e**) PLGA-PEG-PLGA.

Poloxamer is a kind of poly(ethylene oxide)–poly (propylene oxide)–poly (ethylene oxide) (PEO–PPO–PEO) triblock copolymer [53], which is composed of a central hydrophobic poly (propylene oxide) (PPO) core with hydrophilic poly (ethylene oxide) (PEO) chains on both sides [54] (Figure 5a). After heating, the hydrophobic polypropylene oxide group in the poloxamer micelle dries, and when a certain temperature is reached, the micelles begin to contact each other and form a network structure, forming the hydrogel, which is indicated by an increase in viscosity.

Poly (*N*-isopropylacrylamide) is a typical temperature-sensitive material. Its structure contains both hydrophilic amide (-CONH_2_) and hydrophobic isopropyl (-CH(CH_3_)_2_) groups (Figure 5b). At low temperature, many voids exist in the three-dimensional network structure of poly (*N*-isopropylacrylamide). These voids are occupied by water; then, the water molecules form hydrogen bonds with amide groups, and a water molecular layer forms on the surface of the polymer. After the temperature rises, hydrogen bonds are destroyed, isopropyl dehydrates, the water content decreases, the association of hydrophobic groups is strengthened, water is discharged, and the gel contracts. However, because of its poor biodegradability and mechanical properties, this thermosensitive hydrogel has limited applications, usually by introducing other polymers to form interpenetrating polymer networks (IPNs). A poly (*N*-isopropylacrylamide)/polyvinylpyrrolidone (PNIPAM/PVP) interpenetrating polymer network was constructed by introducing polyvinylpyrrolidone. The introduction of PVP shortened the temperature response time of the hydrogel but did not change the critical dissolution temperature (LCST). The drug loading rate of the model drug increased, and the release mode of the drug changed [55].

Cellulose derivatives are biological macromolecules with glucose molecules connected by *β*-1,4-glycosidic bonds (Figure 5c). Cellulose molecules contain many hydrogen bonds, so cellulose temperature-sensitive materials result in a simple entanglement around hydrophobic groups without polymerization at low temperature. After the temperature rises, hydrogen bonds are destroyed and gradually lose their hydration. The strong hydrophobic interaction between cellulose molecules leads to the formation of three-dimensional networks [56], which then form a temperature-sensitive hydrogel. Cellulose-based thermosensitive hydrogels are widely used as biomedical materials because of their nontoxicity, good biocompatibility, and biodegradability. Methyl cellulose and hydroxypropyl cellulose are representative polymers used in cellulose-formed thermosensitive hydrogels.

Chitosan is a natural alkaline amino polysaccharide prepared by deacetylation of chitin, which widely exists in nature. It is composed of an *N*-acetylglucosamine group and a glucosamine residue connected by a *β*-1,4 glycosidic bond [57] (Figure 5d). Chitosan is widely used in the biomedical field because of its high biocompatibility, low cytotoxicity, and biodegradability. Chitosan alone is not thermosensitive; this is usually achieved by blending with sodium glycerophosphate, resulting in the electrostatic attraction of the amino groups on the chitosan and sodium glycerophosphate. When the temperature increases, electrostatic attractions are destroyed, and the chitosan chain dehydrates to a gel. With a certain temperature rise, chitosan deacetylates, the hydrophilic and hydrophobic components on the chain grafted with highly hydrophobic polymers interact with each other, and the viscosity increases to form a hydrogel.

Polyethylene glycol (PEG) can be mixed with polylactide and glycolide (poly (lactide-*co*-glycolide, PLGA) to form a *b-a-b* triblock copolymer PLGA–PEG–PLGA (Figure 5e). After dissolving in water, it forms spherical micelles with a core–shell structure. Its temperature sensitivity has led to its wide use in drug delivery systems. The temperature sensitivity of the copolymer is controlled by the hydrophilic group (PEG) and the hydrophobic group (PLGA) in the polymer. When the temperature is lower than the phase transition temperature, the hydrophobic PEG core and the hydrophilic PLGA shell form micelles via self-assembly. When the temperature rises to the phase transition temperature, the interaction force between the PEG chains strengthens, the shell of PLGA dehydrates, and the micelle aggregation increases, resulting in the polymer solution forming a hydrogel [58]. The sol–gel transition temperature of a polymer can be controlled by the ratio of PEG to PLGA. With the increase in the hydrophilic group PEG in the molecular chain, the phase transition temperature increases. Conversely, the hydrophobic group PEG increases as the phase transition temperature decreases [45]. Similar structures include poly(ε-caprolactone-*co*-lactide)–poly(ethylene glycol)–poly(ε-caprolactone-*co*-lactide) (PCL–PEG–PCL), poly (ε-caprolactone-*co*-glycolic acid)–poly (ethylene glycol)–poly (ε-caprolactone-*co*-glycolic acid) (PCGA–PEG–PCGA), and poly(D,L-lactide)–poly(ethylene glycol)–poly(D,L-lactide) (PDLLA–PEG–PDLLA, PLEL).

### 2.2. Drug Release Mechanism of Thermosensitive Hydrogel

Generally, the drug release mechanisms of thermosensitive hydrogels include (1) diffusion control, (2) swelling control, and (3) erosion control [59].

**Figure 6:**
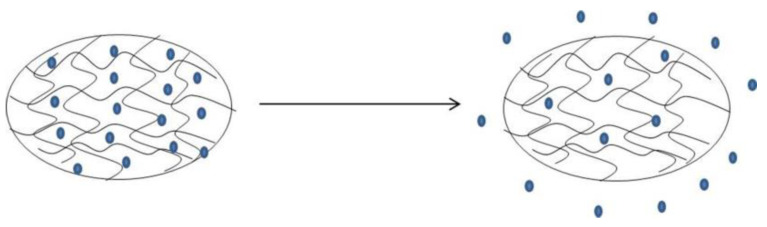
The osmotic pressure between the saturated drug in the gel and the environmental medium, and the drug is released into the medium by free diffusion.

Diffusion control: According to Fick’s first law of diffusion, diffusion control is the most appropriate hydrogel drug release mechanism [60] (Figure 6). A medication system with diffusion control drugs is called a reservoir or matrix system. The diffusion of drugs from hydraulic hydrogels mainly depends on the grid size of the gel matrix, but also on the water radius of the drug molecule. The diffusion is affected by several parameters, mainly including the degree of crosslinking, which constitutes the chemical structure of the single body. When applicable, the type and strength of the external stimulation are also important parameters. Physical properties, such as the mechanical strength, degradability, and diffusion of the hydrogel network, depend to a large extent on its grid size. The typical size of a hydrogel in the expansion state is 5~100 nm, which is larger than most small molecular drugs, so the diffusion of small molecular drugs does not continue. Conversely, macromolecular drugs, such as oligonucleotides, control the release time of protein drugs [61].

**Figure 7:**
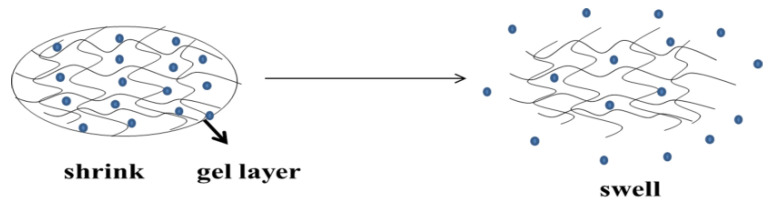
The contracted hydrogel expands in the medium, and the drugs in the swelled gel are released into the medium through the gel layer.

Swelling control: With the expansion control mechanism, when the hydropower radius of the drug molecule is greater than that of the water gel pore, the drug spreads significantly faster than the hydrogel, so the expansion is considered to be controlled [60,61]. When the polymer expands the gel that is in contact with the release medium, the drug passes through the gel layer, so this system is referred to as a bloating control system (nondiffusion-controlled release of a polymer system for drugs (Figure 7)). The swelling force depends on the relaxation and diffusion of the macromolecular chain. When the swelling force is controlled by the macromolecular relaxation process, the drug release does not follow Fick’s diffusion law, which is called case II diffusion or non-Fickian diffusion. In swelling-controlled release systems, the drug is initially dispersed in a glassy polymer, through which the drug cannot diffuse. When the system comes into contact with a thermodynamically compatible release medium (water), the drug is released through the swollen polymer (viscoelastic state) [62].

**Figure 8:**
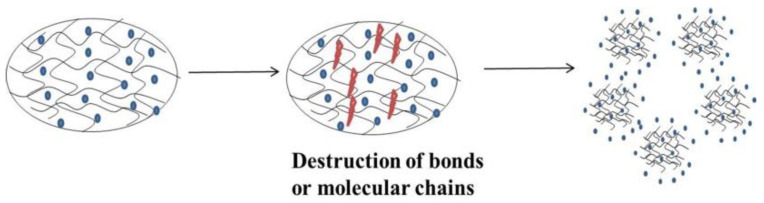
In the medium, the surface of the hydrogel is gradually eroded, the bonds or molecular chains of the internal structure are destroyed, and the drug is released into the medium.

Erosion control: In an eroded hydrogel, the drug is released through the surface or entirely erodes. Eclipse and degradation are the basic aspects of a dissolving hydrogel, where the key or molecular chain is broken, and then the drug is released (Figure 8). This involves a hydrolyzed or enzyme degradation polymer chain or a reversible/irreversible response between the polymer network and the release of the drug. The three mechanisms of polymer erosion are as follows [63]: (1) linking degradation, where the polymer chain is released from the substrate; (2) hydrolysis, ionization, or protonation, which causes the drug to dissolve in water; (3) degradation of unstable primary keys, which generates low-molecular-weight water-soluble molecules. These mechanisms may also be combined [64].

### 2.3. Biodegradation and Adhesion

Hydrogels adhere to the surface of the formed tissue, which is the result of the gel formation process. When using hydrogels, the precursor solution enters the gaps in the tissue surface or its texture, and the precursor solution spreads to the extracellular matrix of the protein and glycosamine. Gelling leads to the stability of two networks: the polymer hydrogel network and tissue texture/tissue protein and glycosaminoglycan network. Because this occurs in the liquid state before stabilization, the two networks are closely entangled. This structure, the interpenetrating polymer network, provides adhesion between polymer hydrogels and tissue bases [65].

For most biological material applications, hydrogel contact with cells and tissues through the surface is inevitable [66]. Therefore, hydrogels for biomedical applications need acceptable biocompatibility and biodegradability. Their hydrophilic surface has low interface free energy when in contact with bodily fluids, which results in a low tendency to adhere to proteins and cells on these surfaces [67]. Because biodegradability is an ideal hydrogel property, a weak bond is often introduced to its structure. Under physiological conditions, this bond is usually destroyed by hydrolysis, which means that the formed compound can metabolize into harmless products or discharge through the kidney filtration process. Block copolymers that form micelles must also be biodegradable because if the molecular weight of the thermal reaction block is lower than the threshold, the polymer obtained by hydrophobic block degradation is rapidly discharged by the kidneys [68].

## 3. Application of Thermosensitive Hydrogel in Local Drug Delivery System

### 3.1. Cancer Treatment

#### 3.1.1. Postoperative Recurrence of Tumor

The clinical treatment of malignant tumors still mainly depends on surgical operation. Although surgery can effectively remove most of the physical tumor tissue, local recurrence and metastasis may also occur [69]. Two factors that cause postoperative tumor recurrence are changes in microcirculation, caused by tumor tissue residues, and surgical trauma, such as the immune microenvironment [70]. For the postoperative administration of adjunctive-related therapeutic agents, routine oral or intravenous administration often requires large doses or repeated dosing to produce the desired therapeutic effect, which can cause side effects or toxicity, can reduce the overall efficacy of the treatment, and can reduce patient compliance [71]. Therefore, alternative strategies are needed to improve tumor administration and reduce systemic drug intake [72]. The partial administration of thermosensitive hydrogels is a promising method because they can release a specific load and control the systemic distribution of therapeutic drugs, thereby improving the expected treatment effect, which reduces the side effects of anticancer drugs [73]. In addition, temperature stimulus is one of the simplest methods with which to accurately control the time and location of the heat response, which can occur naturally and is easy to apply. 

Local recurrence of breast cancer remains a clinical challenge. Camptothecin (CPT) has a strong ability to kill a variety of tumor cells, including breast cancer cells. However, due to its unlimited hydrophobicity, its clinical application has been limited. Early prevention of local recurrence of breast cancer is essential for patients who have received breast-conserving therapy. Wu et al. [74] used CPT to inhibit postoperative recurrence. Hollow mesoporous silica nanoparticles (HMSNs) were used as a carrier (CPT@HMSNs). A PLEL and thermosensitive hydrogel was injected into the tumor site after tumor resection. The recurrence rate and adverse reactions of patients in the CPT group decreased. These hydrogels have unique potential as they can be used as a drug carrier for local transport and are safe to use.

Chen et al. [75] successfully developed a local and long-term Herceptin transfer system for breast cancer to prevent the local recurrence of HER2+ breast cancer after breast-conserving therapy (BCT). Based on an actual blending method, an injectable, temperature-sensitive PLGA–PEG–PLGA mixed hydrogel was prepared, which was loaded with Herceptin. By simply changing the mixing ratio, the hydrogel performance, durability, and drug release curve can be easily changed. A hydrogel matrix with a specific mixing ratio achieved the sustained release of Herceptin in vitro for up to 80 days, which was the longest reported release of it, and the initial explosive release was avoided. A single subcutaneous injection of a hydrogel system can increase the accumulation of tumor antibodies in vivo for up to four weeks, which increases the accumulation of tumor antibodies in vivo. In addition, antitumor and antirecurrence experiments showed that a weekly pulse injection solution can cause cardiac toxicity; however, the continuous release of Herceptin in hydrogel effectively prevents this side effect.

Curcumin (Cur) was encapsulated by liposome and then coated with thiochitosan (CSSH) to form a liposome hydrogel (CSSH/Cur–Lip gel) [76]. This hydrogel is temperature-sensitive and can be injected in situ, leading to rapid gel formation at 37 °C. The cumulative release rate of CSSH/Cur–Lip gel was 31.57% + 1.34% in 12 h, effectively delaying the release of curcumin. With curcumin-encapsulated liposome hydrogel, MCF-7 cells were suppressed and significantly killed after 72 h. An in vivo breast cancer breast cancer recurrence test showed that CSSH/Cur–Lip gel inhibited the recurrence of tumors after breast cancer resection, and the liposome hydrogel continuously transmitted Cur. Mice treated with PBS and CSSH gel had a shorter life span due to tumor growth; Cur–Lip and CSSH/Cur–Lip gel groups had the longest survival of more than 24 days. In addition, no evidence was found of secondary lung metastasis in CSSH/Cur–Lip gel-treated mice, whereas the PBS, Cur, Cur–Lip, and CSSH gel groups all showed lung metastatic nodules.

#### 3.1.2. Cancer Immunotherapy

Surgery can cause local and systemic inflammation, creating an ideal environment for awakening sleeping cancer cells [77]. Unlike traditional treatment strategies, immunotherapy can eliminate local and distant metastatic tumors and can prevent tumor recurrence through long-term immune memory effects [78] through the controlled and sustained release of immunotherapy drugs in tumor parts [79]. The local release of immunotherapy drugs via a polymer hydrogel administration system not only notably improved the effect of immunotherapy but also alleviated immune-related adverse reactions (IRAESs) [80]. The uniqueness of thermosensitive hydrogel systems is that they spontaneously form gels through temperature stimulation and then transfer antigens or vaccines to the target physiological area, thereby improving system efficiency by enhancing immunogenicity and promoting antigen-mediated immunity as an adjuvant. Moreover, hydrogel polymer matrices can degrade in a physiological system [81]. Antitumor immunotherapy vaccine treatment has become an important means to inhibit tumor recurrence and metastases. Injecting vaccines to activate antitumor immunity is effective and safe, avoiding recurrence and metastasis [82]. Injectable smart hydrogels are one of the best candidates for immunotherapy and are safer than systemic administration, as local release of personalized tumor-associated antigens may trigger systemic antitumor immune responses [83].

Circusamide (CTX) not only directly kills tumors, but also immunogenic cells. It is a promising antigen source for cancer vaccines. Yang et al. [84] designed a combined thermosensitive hydrogel (PDLLA–PEG–PDLLA, PLEL) immunotherapy strategy. CTX hydrogel was injected into CT26 mice to start antitumor immunity. Three days later, PLEL hydrogel containing CpG and tumor lysate was injected into both groins to further promote the antitumor immune response. The results showed that the combined strategy reduced the toxicity of CTX, produced cytotoxic T lymphocyte reactions, effectively inhibited tumor growth, extended the time of survival, and significantly increased the tumor cure rate. In addition, about 90% of mice survived the long-term immune response. Only CTX and hydrogel vaccines were needed to achieve a good tumor suppression effect and produce a lasting immune memory response to recurrent tumors. This strategy uses CTX to produce individualized tumors, which induces the original immune response. Then, a tumor-crack vaccine containing tumor-related antigen (TAAS) and a new antigen, to enhance the immune response, were combined with a PLEL hydrogel to form an effective tumor inhibitory effect, improve the survival rate, and reduce recurrence. This thermosensitive hydrogel immunotherapy strategy will promote the commercial production of cancer vaccines and will clinically facilitate the application of chemotherapy and cancer vaccines.

Yang et al. [85] designed an injectable, biodegradable, thermosensitive hydrogel vaccine that can be loaded on GM–CSF, CpG–ODN, and tumor cell lysates (TLs). On the 14th day after administration, compared with the control group, the hydrogel vaccine maintained a higher level of tumor necrosis factor (TNF) in the serum, indicating that it promoted the direct killing of the tumor. In preventive and therapeutic trials, when C26 tumors were immunized by TLs, the hydrogel vaccine significantly delayed tumor growth and prolonged overall survival. Hydrogel vaccines consisting of GM–CSF, CPG–ODN, TLR9 agonists, and tumor cell lysates have important clinical value, as they provide local sustained release antigens and adjuvant activation of dendritic cells.

Lee et al. [86] designed a simple synthetic method based on a PEG polymer gel to introduce carbamate bonds into the block bonding of ABA copolymers. This method formed a gel with higher stability in vivo, so that the protein could be continuously released for >17 weeks and was used to administer cancer prevention vaccines that provided sustained anticancer immunity. The three-ABA-embedded section was arranged into a flower, and the PEG-embedded section was exposed to a water environment. At a higher polymer concentration, a bridging unit was formed between the micelles by inserting the hydrophobic nuclei of the adjacent micelles, which led to the formation of hydrogel networks. During gel formation, OVA was captured in the hydrogel network. Compared with the solution formulation, mice inoculated with hydrogel formulations produced significantly higher antibody levels. Compared with other formulations (12.5~50%), the survival rate of patients treated with OVA with hydrogels and agents was 66.7% higher. This hydrogel is a promising vaccine transport system.

Wei et al. [87] subcutaneously injected thermosensitive hydrogels (DT gels) based on PLGA–PEG–PLGA, which were loaded with a bispecific anticluster of CD3 scFv T–cell/antiepidermal growth factor receptor (EGFR) Fab engager (BiTEE). The temperature-sensitive property of the 28–34 °C sol–gel transition was suitable for injection. The in vitro release experiment showed that all DT gel preparations, which had a stable bite force, extended the time of the BiTEE force to seven days. In an animal pharmacokinetic study, a subcutaneous injection of BiTEE/DT gels prolonged the half-life of BiTEE by 2.2 times compared with intravenous injection. In summary, an injective BiTEE polyethylene glycol PLGA thermosensitive hydrogel was successfully formed that had an extended half-life, maintained a constant blood level in the treatment window, and enhanced T cell presence in solid tumor sites, thereby achieving special therapeutic effects.

### 3.2. Prevention of Postoperative Adhesion

Postoperative adhesions are a common postoperative complication, mostly occurring in the abdomen, joints, and pelvic area, with an incidence of between 50% and 97% [88]. Postoperative adhesion is the natural result of the wound healing of surgical tissue and can cause infertility, pain, or intestinal obstruction [89]. To prevent the formation of adhesion after surgery, hormone therapy and antiadhesive barriers have been applied, which involve the use of mechanical devices or chemicals with anti-inflammatory characteristics, antioxidants, anticoagulants, and fibrin solvents [90]. However, their biological safety is poor, producing many adverse reactions, and the biological adhesion is insufficient. Mechanical devices can easily fall off of the action site. Barriers have poor viscosity and poor coverage of postoperative wounds, especially for irregular wounds. The reserved part of the role was too short and degraded during the critical period of adhesion that occurred after surgery. Thermosensitive hydrogels are flowing liquids at low temperature, have good liquidity, are easy to use, and therefore can cover humid and irregular wounds. Under the influence of body temperature, thermosensitive hydrogels quickly bond and attach to the area, and their biodegradation speed can be controlled. During the critical postoperative period, the adhesion does not degrade. These advantages have led to thermosensitive hydrogels attracting widespread attention in antiadhesion research in the field of surgery.

Chou et al. [91] evaluated the in situ thermal response of a hydrogel based on poly (*N*-isopropyl acrylamide) (PNIPAM) to prevent postoperative adhesion around an aponeurosis. The hydrophilic biopolymer chitosan (CS) and hyaluronic acid (HA) were grafted onto PNIPAM, and the copolymer hydrogel exhibited enhanced water retention and lubricity during phase transition and reduced volume shrinkage during phase transformation. In the cell culture experiment, the thermosensitive hydrogel showed good biocompatibility and reduced the permeability of fibroblasts. In animal experiments, the rabbit deep flexor tendon model was adopted to study the effectiveness of the periodontal adhesion of the postoperative tendon. From general inspections, histology, the joint bending angle, tendon slip, and stretching force, PNIPAM and CS–PNIPAM (CPN) hydrogels, the commercial barrier film Seprafilm^®^, and HA–CS–PNIPAM (HACPN) were found to prevent the need for surgery. The best barrier material for rear tendon adhesion was MISSING. After treatment with the HACPN hydrogel, the tendon′s fracture strength was not significantly different from that of the self-healed tendon, indicating that the application of the HACPN hydrogel did not affect the normal healing of the tendon. The clinical advantage of thermosensitive hydrogels is that, as a barrier material, they can prevent fibroblasts from penetrating, provide liquidity and flexibility during the application process, and can fill any shape or space with only a small opening injection.

Similarly, Chen et al. [92] grafted poly (*N*-isopropylacrylamide) (PNIPAm) onto chitosan (CS) and further coupled the polymer with hyaluronic acid (HA) to form a thermosensitive hydrogel, HA–CS–PNIPAm. HA–CS–PNIPAM aqueous solution flows freely and can be injected at room temperature, and the sol–gel phase changes at about 31 °C. Cell culture research showed that hydrogels provide a good barrier function in vitro and can therefore reduce the induction of cytotoxicity and the penetration of fibroblasts. In the rat sidewall defect intestinal wear model, the antiadhesion effect of the HA–CS–PNIPAM hydrogel was better than that of PNIPAM and the unprocessed control group. Two weeks after treatment, no abdominal interruption was observed with scanning electron microscopy. Histological and blood tests supported the lack of either organ injury or acute toxicity. This common polymer hydrogel uses HA–CS–PNIPAm′s easy operation and application processes and is therefore an ideal material to be injected as an antiadhesion barrier after abdominal surgery.

In a randomized, controlled, single-blinded clinical trial conducted by Kim et al. [93], the subjects were women who underwent benign gynecological surgery from January to December 2017. Patients were randomly divided (1:1:1) into a test group (received thermosensitive hydrogel treatment), control group (nontreatment), or a comparator group (received 4% icodextrin treatment), with four weeks of follow-up. They evaluated the formation of adhesion by a visceral sliding test. The incidence of abdominal adhesion in the experimental group was significantly lower than that in the control group (7.9% vs. 21.1%, *p* = 0.040), and the incidence in the experimental group was lower than that in the comparison group (7.9% vs. 13.8%, *p* = 0.299). In this clinical trial, patients who underwent gynecologic pelvic surgery showed decreased adherence formation with the use of a poloxamer-based thermosensitive hydrogel compared with patients who did not receive antiadhesive therapy, and no safety issues were noted. Surgeons were significantly more satisfied with the treatment with this drug than with the use of 4% icodextrin solution.

### 3.3. Nasal Brain Targeting

When a traditional liquid preparation is administered, the rapid removal of mucosal cilia can lead to short retention times and poor biological use. Compared with liquid nasal cavity preparations, nasal cavity gels are dripped into the nasal cavity with a low viscosity solution. After the hydrogel is in contact with the nasal mucosa, the construct of the gel can be changed, so the drug is retained on the nasal mucosa and the drug is continuously released. The nasal cavity has special physiological characteristics. The drugs that are administered through the nasal cavity bypass the blood–brain barrier through inhalation and trigeminal neuropathy and directly and effectively transfer the treatment to different areas of the brain, successfully transmitting drugs to the brain [94]. In situ hydrogel therapy increases the nasal retention time, reduces the removal of mucus cilia and the degradation of enzymes, and overcomes the limitations of direct nasal to brain transportation. This medication system further improves the biological use of the nasal cavity and drugs in the brain.

Neurological diseases include Parkinson′s disease, epilepsy, multiple sclerosis, Alzheimer′s disease, cerebrovascular disease, and brain tumors. The methods of treating these diseases include local, oral, and intravenous injection treatment; device-oriented or equipment-based treatment; and methods such as deep brain stimulation, hand surgery, and recovery. Other methods include injection into the brain or cerebrospinal fluid or intranasal injection. Some of these technologies are unsafe, invasive, and local, with short-term effects. The in situ hydrogel system can bypass the blood–brain barrier, transport drugs to the required parts, reduce peripheral toxicity, and offer control of the drug release dynamics [95].

For Parkinson′s disease treatment, Wang et al. [96] designed a polymer micelle temperature-sensitive gel (ROT–PM–TSG) delivery system for rothgatin (ROT) to improve the solubility of the drug, prolong its residence time, and increase its concentration in brain tissue. First, the ROT–loaded polymer micelles (ROT–PMs) were customized and optimized. The average particle size, entrapment efficiency, and drug loading of ROT–PM were 88.62 ± 1.47 nm, 93.5% ± 0.79%, and 19.9% ± 0.60%, respectively. The best ROT–PM–TSG formula contained 22% P407 and 2% P188, the gel temperature was about 32.3 °C, and the pH was 5.186. In vivo, the MRT prolongation of ROT–PM and ROT–PM–TSG by nasal administration was 1.43 and 1.79 times that of intravenous injection, respectively. Compared with the intravenous injection group, the distribution proportions of ROT in the olfactory bulb, brain, cerebellum, and striatum were 276.6%, 170.5%, 166.5%, and 184.4%, respectively. In conclusion, the ROT–PM–TSG system is a suitable ROT nasal brain delivery system and has application prospects.

Although opioids have a strong analgesic activity, their effect after intravenous injection may be due to the rapid degradation of peptidase in the blood, which may seriously hinder the successful application of opioids in clinical applications. To overcome this problem, Mura et al. [97] developed an opioid–liposome-adherent thermosensitive hydrogel, which showed a protective effect on drugs and significantly improved the analgesic effect range and duration. The optimized hydrogel formula was based on a P407 (26.5%) and carbomer (1%) combination, which shortened the gelation time; that is, the nasal application time (10 s), gel temperature (33.7 °C), and gel strength were shown to be suitable. In in vitro penetration experiments, through the removal of porcine nasal mucosa, the developed hydrogel formulations provided the sustained and controlled delivery of drugs over 5 h, emphasizing the importance of the role of liposome vectors by increasing the permeability coefficient and permeability six times through the lipophilic nasal mucosa. Good adhesion and a long adhesion time are important for liquid formulations, and the rapid gel process ensures a prolonged residence time, which is beneficial for drug absorption.

Oral antidepressants have many side effects such as dizziness, diarrhea, and drug resistance. Therefore, new antidepressants and natural drug derivatives are required. In a pill form, berberine (BBR) and EVO were combined to treat depression. Xu et al. [98] prepared a self-assembled, temperature-sensitive hydrogel to achieve continuous cotransmission of BBR and EVO to treat depression. They encapsulated BBR and EVO into HP-β-CD to improve the solubility and bioavailability of the drugs and combined hydroxypropyl-β-cyclodextrin (HP-β-CD) with poloxamer to construct a thermosensitive hydrogel, which showed good temperature sensitivity. The antidepressant effect of the hydrogel was studied by the behavioral despair model and the reserpine-induced model, and the antidepressant mechanism was analyzed by omics. In the in vivo experiments, intranasally injected hydrogels were rapidly absorbed into the brain, significantly increasing the bioavailability of BBR and EVO in vivo. The low-dose hydrogel significantly reversed behavioral despair in mice, alleviated the depressive symptoms induced by reserpine, and improved the abnormal levels of 5-HT, NE, DA, and other monoamine neurotransmitters in the rat brain. The findings of metabolic studies in rat hippocampus suggested that hydrogel administration may improve depression by regulating ascorbic, Alda, and butyric acids; vitamin B6; and pyrimidine. These results indicated that hydrogel, when used as a local and codelivery carrier, can modulate monoamine neurotransmitters in those with depression.

### 3.4. Wound Healing

Various types of formulas, such as water gels, films, foams, stents, particles, and nanoparticles have been used as wound healing materials. Among them, hydrogels are one of the most commonly used systems for treating acute and chronic wounds. Hydrogels are composed of hydrophilic polymers and enable water absorption and secretions [99]. The water supply effect of hydrogels helps them to increase the production of collagen enzymes, increase the water content of necrotic wounds, and enable self-soluble debridement [100]. Because of their excellent temperature sensitivity and injection characteristics, hydrogels are suitable for loading with cells or drugs and can fill any irregular wound parts [101]. As a free flowing liquid before low-temperature gelation, a hydrogel precursor solution can be injected into the wound area, which quickly fills irregular defects and forms gels under physiological conditions.

Lan et al. [102] developed a hybrid hydrogel dressing for locating and continuously delivering MMP-9 siRNA (siMMP-9). SiMMP-9 was compounded with Gly–TETA (GT), and then the GT/siMMP9 complex was loaded onto thermosensitive hydrogel based on pluronicF-127 (PF) and methylcellulose (MC). SiRNA has considerable therapeutic potential because of its precise mode of action and its ability to rapidly silence targeted gene expression. The results of an in vivo evaluation of diabetic rats showed that the hydrogel released GT/siMMP9 into the wound tissue through temperature-sensitive control for seven days, providing local and continuous transmission, resulting in significant silencing of MMP-9 and an improvement in the closure of diabetes wounds. This mixed hydrogel dressing had good biocompatibility and did not produce systemic toxicity in rats. In summary, hybrid hydrogel dressings may constitute biocompatible materials that effectively promote the treatment of diabetes by effectively silencing the MMP-9 gene, providing a platform for siRNA applications in other diseases.

Various types of mesenchymal stem cell-derived exosomes (MSc-exos) have a variety of therapeutic effects on chronic refractory wounds caused by diabetes. Yang et al. [103] successfully constructed a composite of human umbilical cord mesenchymal stem cell (hUCMSC-exos)-derived exosomes and a pluronicf-127 (PF-127)-thermosensitive hydrogel to treat chronic diabetes wounds. The PF-127 thermosensitive hydrogel is suitable for carrying and continuously releasing exosomes to promote the formation of wound endothelial cells. Both in vivo and in vitro, the application of the exosome hydrogel composite enabled the continuous release of biological molecules such as exosomes, stimulated the early angiogenesis of diabetes wounds, and promoted wound healing and skin remodeling.

Thermosensitive hydrogels can be used as drug carriers for local delivery and can activate immune cells to promote wound healing. Andrgie et al. [104] used loaded ibuprofen on heparin-coupled poly (N-isopropylacrylamide), an injectable in situ gel-forming polymer, to help reduce pain and excessive inflammation during healing. They used the BALB/c mouse model to evaluate its effects on inflammatory healing and secretion of inflammatory mediators. An in vivo wound-healing experiment was conducted by applying the hydrogel to a wound on the back of mice. The results showed that the ibuprofen-loaded hydrogel improved healing compared with a phosphate-buffered saline group. The results of in vitro experiments confirmed that the ibuprofen released by the hydrogel inhibited RAW264.7 in macrophages, NO, pge2, and TNF-α. The production of lipopolysaccharides significantly reduced the inflammatory response induced by lipopolysaccharides. Injectable ibuprofen hydrogel is a promising treatment for wound healing.

### 3.5. Osteoarthritis

Osteoarthritis (OA) is one of the most common chronic degenerative arthritis diseases. The main pathological features of OA include cartilage degeneration, subchondral bone remodeling, and synovial inflammation [105]. Thermosensitive hydrogels are liquid when administered, and the polymer materials in the articular cavity respond to temperature to form a semisolid gel. They can prolong the release and action time of therapeutic drugs, have high drug stability, and can be targeted to the articular cavity [106]. Intra-articular treatment is a more effective method for osteoarthritis at present, as the initial effect of oral drugs is prevented, and the side effects of systemic poisoning are avoided.

With chitosan–glycerin–borax as the carrier, Wang et al. [107] used medial meniscus (DMM) instability in mice to illustrate the effect of a new intra-articular (IA)-loaded dexamethasone temperature-sensitive hydrogel (DLTH) on the inhibition and pain relief of osteoarthritis (OA). This in situ DLTH formed at 37 °C in the articular cavity. The cumulative release curve of dexamethasone (DEX) at 37 °C showed that DLTH was rapidly released in the first 24 h and continued to be slowly released for seven days. The results of in vivo studies showed that DLTH can reduce DMM-induced bone destruction in mice and slow the progression of synovitis and OA. DLTH had an analgesic effect on OA mice, shortened the nociceptive response time (NRT), and downregulated serum PGE2, inflammatory factors, and pain-related mediators.

Intra-articular flurbiprofen is often used to treat osteoarthritis, but due to the limited effective time, repeated injections are required. To improve the treatment effect and prolong the treatment interval, Li et al. [108] studied the suitability of PCLA–PEG–PCLA, a triblock flurbiprofen copolymer and thermosensitive hydrogel, for continuous intra-articular administration. They studied the antiosteoarthritis effect of this hydrogel in a collagenase-II-induced rat knee osteoarthritis model, and the results were compared with those obtained in response to routine sodium hyaluronate and flurbiprofen injection. The results of in vitro drug release studies showed that flurbiprofen was continuously released from the thermosensitive hydrogel for more than three weeks. In addition, compared with flurbiprofen solution, flurbiprofen hydrogel treatment significantly improved the final total ipsilateral paw print intensity (%TIPPI) and decreased the knee bending score. These results showed that flurbiprofen in a thermosensitive hydrogel maintains its analgesic and anti-inflammatory activities through sustained drug release, and the use of the hydrogel substantially improved the long-term analgesic effect. In the flurbiprofen-hydrogel-treated group, the inflammatory response weakened and the levels of IL-1, IL-6, and IL-11 in joint fluid decreased. The temperature-sensitive copolymer PCLA–PEG–PCLA was found to be suitable for sustaining the intra-articular effect of flurbiprofen and provided an experimental basis for potential clinical applications of flurbiprofen hydrogels to improve the management of patients with OA.

Glucosamine (GlcN) is a drug commonly used for the treatment of osteoarthritis (OA). To prolong the action time of glucosamine and improve its therapeutic effect, Zhang et al. [109] discussed the application potential of a GlcN-loaded thermosensitive hydrogel based on poloxamer 407 and poloxamer 188 for the treatment of OA. The thermosensitive hydrogel was prepared using a cold method. After screening, the optimized formula showed good temperature sensitivity, and the gelling temperature was approximately 35 °C. The results of the in vitro release test showed that GlcN was slowly released from the thermosensitive hydrogel through passive diffusion and gel dissolution. After intra-articular administration of the hydrogel to treat OA in rabbits, the degree of swelling and inflammatory factors in the hydrogel-treated group significantly decreased (*p* < 0.05). From the histological results, the cartilage repair effect in the GlcN-treated group was good. At the same dosage, the treatment effect of the temperature-sensitive hydrogel was better than that of the aqueous solution. Therefore, the GlcN-loaded thermosensitive hydrogel based on poloxamer was found to be a promising and sustainable drug delivery system for intra-articular injection therapy.

Thermosensitive hydrogels are unique biomaterials that have shown considerable potential in maintaining drug release. Many efforts have been devoted toward increasing stimulation sensitivity and adding hydrophilic molecules to a stable hydrogel network. The formulation of biodegradable and stable thermosensitive hydrogels is still being extensively studied. The development of drug delivery systems based on thermosensitive hydrogels using polymers with different structures and physical and chemical properties is increasingly attracting attention in the scientific community because these systems show the ability to be used to treat a wide range of various diseases. More research examples are listed in Table 1.

## 4. Conclusions

Thermosensitive hydrogels can change their state with changes in ambient temperature. This special advantage can prolong the residence time of a drug in the body, increase the local drug concentration, improve drug bioavailability, enable direct administration at the disease site, and expand the scope of drug applications. Thermosensitive hydrogel drug delivery systems have attracted increased research attention in biomedicine, and the efficacy of many of these preparations has been verified in animal models. However, in the next step in realizing hydrogel applications in the human body, some aspects need to be further studied, including the viscosity, mechanical strength, rheological properties, stability, and in vitro release methods of thermosensitive hydrogels; the physiological stability, compatibility, biodegradability, pharmacology, and toxicology of the materials must be understood in detail. However, with research advances, more thermosensitive hydrogel preparations will be used in clinical medicine, bioengineering, tissue engineering, and other fields, as they have broad application prospects.

## Figures and Tables

**Table 1:** Application of thermosensitive gel constructed by thermosensitive materials in biomedicine.

Classification	Characteristic	Polymer	Drug and Application	Objective of the Study	Reference
Natural	Excellent biocompatibility, low toxicity, good compatibility with other chemical reagents and strong solubilization, which can delay drug release	Poloxamer407/188	Almotriptan malate	Deliver drugs through the nose to the brain	[110]
		Poloxamer407/188	Voriconazole	Vaginal infection	[111]
		Poloxamer407	Doxorubicin	Antitumor and antiangiogenic efficacy	[112]
		Pluronic^®^F127	OMV-antigenic complex	Intranasal delivery system of bacterial antigen	[113]
		Poloxamer407	Amikacin	Accelerated wound healing	[99]
		Pluronic^®^F127 oxidized hyaluronic acid	/	Postoperative antiadhesion barrier	[114]
		Poloxamer407	Doxorubicin and ICG.	Prevention of local tumor Recurrence after surgery	[115]
		Poloxamer 407 and hyaluronic acid	keratinocyte growth factor 2	Knee osteoarthritis	[116]
		Poloxamer407/188	Desloratadine	Antiallergic agent through the nose	[117]
	Natural linear polymer, high porosity, biodegradable, nontoxic, antibacterial, good biocompatibility, good temperature sensitivity and antibacterial hemostatic properties, has a powerful function in promoting wound healing	Chitosan	Methotrexate	Controls tumor cell growth	[118]
		Chitosan	/	Sealing and lubricating purposes in dental implant system	[119]
		Chitosan	Recombinant human collagen-peptide (RHC)	Cell encapsulation and wound repair	[119]
		Chitosan	Tranexamic Acid	Localized treatment of nasal wounds	[120]
		Chitosan	Gallic acid	Wound healing	[121]
		Chitosan	Ferulic acid	Peripheral arterial disease	[122]
		Poloxamer-chitosan	Vitamins A, D and E	Skin burns	[123]
	Natural-derived polymers, through the introduction of hydrophobic groups through chemical modification, make cellulose have temperature-sensitive properties. Different synthetic polymers are mixed with cellulose to adjust its drug release characteristics. They are biodegradable and biocompatible	Cellulose	carbon-based nanozyme	Antibacterial application	[124]
		Methylcellulose	Bone mesenchymal stem cells	Bone regeneration	[125]
		Poloxamer 407, sodium carboxymethyl cellulose, chitosan	Benzydamine hydrochloride	Oral mucosa diseases	[126]
		Methyl cellulose (MC), Hyaluronic acid (HA)	/	Postsurgical de novo peritoneal adhesion	[127]
		Poloxamer 407, chitosan (CS), methyl cellulose (MC)	L-carnosine	Wound healing effect	[128]
Synthesis	Polyethylene glycol has very high hydrophilicity and can crosslink degradable polyester at the end to obtain multi block copolymer. The change of molecular weight and block ratio of the copolymer can realize the intelligent adjustment of the hydrophilic and hydrophobic properties and temperature sensitivity of the material	PLGA–PEG–PLGA	Ropivacaine Hydrochloride	Postoperative pain relief	[129]
		PLGA–PEG–PLGA	Dexamethasone	Alkali-burn-induced corneal neovascularization	[130]
		Pluronic^®^F127, PLGA–PEG–PLGA	Salinomycin	Glioblastoma therapy	[131]
		PCL-PEG-PCL	Diclofenac sodium	Anti-inflammatory and analgesic	[132]
		PLGA-PEG-PLGA	Curcumin, Doxorubicin	Localized administration for osteosarcoma	[133]
		PLGA-PEG-PLGA	Corilagin, chitosan	Localized cancer therapy	[134]
	Good temperature sensitivity, simple preparation, easy availability of materials, numerous modified monomers, crosslinking with a variety of temperature-sensitive materials, and rich functional properties	poly(*N*-isopropylacrylamide)	Cell	High-resolution bioprinting	[134]
		poly(*N*-isopropylacrylamide)	/	Immunoglobulin G (IgG) purification	[135]
		poly(*N*-isopropylacrylamide)	Diclofenac sodium (DS)	Skin reinfection	[136]
		poly(*N*-isopropylacrylamide)	Brown adipose-derived stem cells	Stem cell transplantation in myocardial repair	[137]
		Sodium alginate-g-poly (*N*-isopropylacrylamide)	Curcumin	Wound healing	[24]
		poly(*N*-isopropylacrylamide)	Wharton′s jelly-derived mesenchymal stem cells	3D cell carriers for regenerative medicine	[138]
		poly(*N*-isopropylacrylamide)	Dextran	Postinfarct heart failure After degradation	[139]

## Data Availability

Not applicable.
